# Notes and News

**Published:** 1892-11-19

**Authors:** 


### NOTES AND NEWS.

COLLECTED AND COMMUNICATED.

WE were much alarmed recently to learn that "a great explosion of scarlet fever" had taken place at Glasgow. Such a species of catastrophe sounds so much worse than an "outbreak." Perhaps the fact that the expression was used at a meeting of Police Commissioners accounts for its strength.

THE New Hospital for Women, Euston Road, makes a special appeal to ladies for help towards this work amongst their own sex. From the commencement the undertaking has been most successful, and greatly appreciated. The original number of beds was ten; these have now been increased to forty-two, and have been continuously occupied. The medical staff is entirely female, and the hospital affords an admirable education to women studying to minister to their own sex. The hospital, therefore, has special claims upon women, and when it is known that over £1,700 is necessary to supplement the reliable income of the institution, we feel confident that ladies will come forward with generous assistance.

THE usual visitation of the Dublin Hospital by the Corporation Committee took place recently. The Committee reported favourably on nearly all of the institutions, faults only being found at the Coombe Lying-in Hospital, where the cleanliness in the chronic department was not found to be satisfactory; and at the National Lying-in Hospital, where the premises were condemned as unsuitable. In the City of Dublin Hospital the food supplied to the patients was noted as excellent. The wards in St. Vincent's Hospital were found to be "scrupulously clean." The cooking arrangements and laundry at the Hospital for Incurables were much commended, as was also the loving ca e shown to the inmates of the Hospice for the Dying. Many important changes in various institutions were recorded since the last visitation. There can be no doubt that these periodical inspections tend to stimulate the efforts of the Dublin charities and increase their efficiency.

WE were a little startled on perusing the columns of a contemporary to come across the announcement that the splendid new Clarence wing at St. Mary's Hospital was now completed, and about to be opened by the Princess of Wales in December. We don't ourselves see how it could be very much more "open," but the rest is rather more than poetic or even journalistic licence justifies.

WE are requested to announce that a Post-graduate course of Lectures will be held at the Central London Throat, Nose, and Ear Hospital, Gray's Inn Road, during the present winter, by Mr. Lennox Browne and Dr. Dundas Grant. Mr. Lennox Browne will lecture on the Nares, Pharynx, and Larynx, commencing Monday, November 14th, and subsequent Mondays, at five o'clock. Dr. Dundas Grant will lecture on Diseases of the Ear, commencing November 16th, and on subsequent Thursdays, at five o'clock. These lectures are open to qualified practitioners and fourth year students on payment of a fee of one guinea.

THE appeal for a reinstitution of the grant of £250 per annum formerly received by the Rotunda Hospital from the Dublin Corporation, has caused a good deal of "feeling" and some mud-throwing. The matter has revolved round two questions—that of sect, and that of the master's emoluments. The Catholic papers point out that the majority of the management is Protestant, and that no post of value is practicably open to Catholics. The Protestant papers draw attention to the fact that the larger amount of subscriptions come from the Protestant community, whilst the majority of patients are Catholic. To this Mr. T. Sullivan, M.P., bore witness, and testified also to the fairness of the Protestant management. He was the only Catholic present at the meeting of the Corporation to consider the appeal for the grant, who voted for the motion, which was lost by an overwhelming majority.

AT St. George's Hospital, every year, an interesting little exhibition takes place which, although not widely known, is nevertheless much appreciated by those who visit it both in and outside the hospital. The St. George's Hospital Graphic Society was called into existence in 1889, under the presidency of the late Sir Prescott Hewett, for the purpose of encouraging photography, wood carving, and art in general, amongst past and present St. George's men. The last exhibition was held on November 2nd, and during the succeeding week. That the intention of the society has been fulfilled was shown by the excellency and number of the exhibits. Amongst these were two charming water-colours by the late president. Dr. Coulthard's, "Head of a Child," in oils; "Old Windsor," by Dr. Blandford, and "Basingstoke Canal," by J. Hawes, were amongst the most admired works of art. There were many admirable photographs, and some good pen and inks. Altogether, the little society is worthy of warm encouragement. Its aims tend towards broadening and uniting the interests of the little world of St. George's, and we cannot doubt but that such a society would be equally popular in other institutions which might have enterprise enough to start one.

